# Arrhythmic risk stratification in heart failure mid‐range ejection fraction patients with a non‐invasive guiding to programmed ventricular stimulation two‐step approach

**DOI:** 10.1002/joa3.12416

**Published:** 2020-08-02

**Authors:** Petros Arsenos, Konstantinos A. Gatzoulis, Ioannis Doundoulakis, Polychronis Dilaveris, Christos‐Konstantinos Antoniou, Soulaidopoulos Stergios, Skevos Sideris, Sotiropoulos Ilias, Dimitrios Tousoulis

**Affiliations:** ^1^ First Department of Cardiology and Electrophysiology Laboratory Hippokration General Hospital National and Kapodistrian University of Athens School of Medicine Athens Greece; ^2^ Arsenos Heart and Biosignals Lab Avlonas Greece; ^3^ State Department of Cardiology Hippokration General Hospital Athens Greece

**Keywords:** arrhythmic sudden cardiac death, mid‐range ejection fraction heart failure, non‐invasive risk factors, programmed ventricular stimulation, two‐step risk stratification approach

## Abstract

**Background:**

Although some post myocardial infarction (post‐MI) and dilated cardiomyopathy (DCM) patients with mid‐range ejection fraction heart failure (HFmrEF/40%‐49%) face an increased risk for arrhythmic sudden cardiac death (SCD), current guidelines do not recommend an implantable cardiac defibrilator (ICD). We risk stratified hospitalized HFmrEF patients for SCD with a combined non‐invasive risk factors (NIRFs) guiding to programmed ventricular stimulation (PVS) two‐step approach.

**Methods:**

Forty‐eight patients (male = 83%, age = 64 ± 14 years, LVEF = 45 ± 5%, CAD = 69%, DCM = 31%) underwent a NIRFs screening first‐step with electrocardiogram (ECG), SAECG, Echocardiography and 24‐hour ambulatory ECG (AECG). Thirty‐two patients with presence of one of three NIRFs (SAECG ≥ 2 positive criteria for late potentials, ventricular premature beats ≥ 240/24 hours, and non‐sustained ventricular tachycardia [VT] episode ≥ 1/24 hours) were further investigated with PVS. Patients were classified as either low risk (Group 1, n = 16, NIRFs−), moderate risk (Group 2, n = 18, NIRFs+/PVS−), and high risk (Group 3, n = 14, NIRFs+/PVS+). All in Group 3 received an ICD.

**Results:**

After 41 ± 18 months, 9 of 48 patients, experienced the major arrhythmic event (MAE) endpoint (clinical VT/fibrillation = 3, appropriate ICD activation = 6). The endpoint occurred more frequently in Group 3 (7/14, 50%) than in Groups 1 and 2 (2/34, 5.8%). Logistic regression model adjusted for PVS, age, and LVEF revealed that PVS was an independent MAE predictor (OR: 21.152, 95% CI: 2.618‐170.887, *P* = .004). Kaplan‐Meier curves diverged significantly (log rank, *P* < .001) while PVS negative predictive value was 94%.

**Conclusions:**

In hospitalized HFmrEF post‐MI and DCM patients, a NIRFs guiding to PVS two‐step approach efficiently detected the subgroup at increased risk for MAE.

## Graphical abstract

We applied a combined non‐invasive risk factors (NIRFs) leading to programmed ventricular stimulation (PVS), a two‐step risk stratification algorithm, to detect the HFmrEF/40‐49% patients at risk for major arrhythmic events.This combined two‐step approach efficiently detected the patients at increased arrhythmic risk.

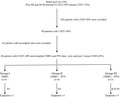

## 1 INTRODUCTION

Heart failure (HF) with mid‐range ejection fraction 40%–49% (HFmrEF) was introduced in 2016 by the task force for the diagnosis and treatment of acute and chronic HF of the European Society of Cardiology^1^ as an intermediate phenotype between HF with reduced ejection fraction < 40% (HFrEF) and HF with preserved ejection fraction > 50% (HFpEF). Evidence emerging from epidemiological studies suggest that arrhythmic sudden cardiac death (SCD) is a frequent mode of death for both HFmrEF and HFpEF patients.^2^, ^3^ Annual SCD rates observed in CHARM‐Preserved^4^ (n = 3022, left ventricular ejection fraction [LVEF] >40%), in TOPCAT^5^ (n = 1767, LVEF > 45%) and in Ikeda's^6^ (n = 1041, LVEF = 55%) studies were 1.4%, 1.4%, and 0.6%, respectively. In the PRE‐DETERMINE^7^ study, the HFmrEF subgroup (n = 1756, LVEF 40%–49%) had annual SCD rate 0.8%. Such SCD frequency may justify arrhythmic SCD as a possible therapeutic target but lack of methods for the detection of the truly HFmrEF high arrhythmic risk patients lead these patients to be ignored by the current guidelines which recommend an implantable cardiac defibrilator (ICD) only for those with a depressed LVEF ≤ 35%.^8^ Furthermore, these populations are heterogeneous with frequent comorbidities including hypertension, coronary artery disease, atrial fibrillation, cerebrovascular disease, obesity, and anemia.^9^ Hence, these patients with competing risks demand the adequate directed toward the arrhythmic risk approaches.

Previous attempts for SCD risk estimation of patients with relatively preserved left ventricular systolic function were based on prognostic scores derived from clinical characteristics,^10^ such as the presence of left bundle branch block, increased natriuretic peptide levels^11^ and impaired glomerular filtration rate.^12^ These initial prognostic models had a low sensitivity for the arrhythmic SCD occurrence.

We recently published results from the PRESERVE‐EF study^13^ addressing post‐myocardial infarction (post‐MI) patients with well preserved left ventricular systolic function and a mean LVEF 50.8% for the risk of arrhythmic SCD. Our two‐step algorithm (non‐invasive risk factors [NIRFs] guiding to PVS) detected efficiently the subpopulation at risk for major arrythmic events (MAE) that could be protected by an ICD. Thus, in the present study, we investigated post‐MI and dilated cardiomyopathy (DCM) HFmrEF patients with a mean LVEF = 45% for their SCD risk with a combined NIRFs leading to a PVS two‐step approach. Patients found at risk received an ICD for primary prevention of SCD and the results of their long‐term follow‐up are presented.

## 2 METHODS

### 2.1 Patients and study design

The study conforms with the principles outlined in the Declaration of Helsinki. It was approved by our Institution's Ethics Committee and 416 post‐MI and DCM patients hospitalized in our Cardiology Department between 2005 and 2013 were included after providing informed consent. All patients were referred to our EP Lab for risk stratification for primary SCD prevention. Patients with an impaired left ventricular systolic function (LVEF ≤50%) were included. Coronary artery disease (CAD) was confirmed by coronary angiography following angina, non ST elevation‐MI and ST elevation‐MI while DCM diagnosis was based on clinical, echocardiographic, and angiographic findings in the presence of systolic left ventricular dysfunction of undefined pathogenesis. Patients with ongoing myocardial ischemia anticipated to be improved with revascularization, acute myocarditis, significant valvular disease, hypertrophic or restricted cardiomyopathy, chronic atrial fibrillation, alcohol‐associated disease, cardiac toxicity, malignant diseases affecting survival, end‐stage renal failure on hemodialysis were excluded. The screening was performed in two‐steps. In the first step, patients underwent a NIRFs investigation with electrocardiogram (ECG), signal averaged ECG (SAECG), 24‐hour ambulatory ECG (AECG), and echocardiography (ECHO), and in the second one, those found with at least one positive NIRF were submitted to PVS.^14^ Although European^8^ and American^15^ guidelines for ICD implantation do not routinely recommend PVS for risk stratification, according to the national guidelines in Greece, induction of sustained ventricular tachycardia (VT)/ventricular fibrillation (VF) is usually required to approve ICD implantation for primary prevention of SCD regardless of the severity of co‐existing left ventricular dysfunction. Previously published data support PVS use for risk stratification in both post‐MI^16^, ^17^ and DCM^18^ patients with well‐preserved LVEF.

### 2.2 The combined algorithm of non‐invasive risk factors presence guiding to programmed ventricular stimulation two‐step approach

#### 2.2.1 First‐step: Non‐invasive risk factors screening

Non‐invasive risk factors criteria from SAECG and 24‐hour AECG for electrical instability predisposing to possible future malignant arrhythmias included:

1. Presence of Late Potentials diagnosed if 2 out of 3 conventional^19^ or modified^20^ criteria were present (acceptable noise level < 0.6 μV).
2. One or more non‐sustained ventricular tachycardia (NSVT) episodes^21^ (at least three QRS complexes at a rate of ≥ 100 beats per minute) on 24‐hour AECG.
3. Ventricular premature beats ≥ 240/24 hours^22^ on 24‐hour AECG.

Patients with presence of one positive out of these three criteria were further stratified with PVS in the second‐step.

##### Echocardiography

A complete ECHO examination was performed (SONOS 5500, Hewlett Packard, Andover, MA,USA). LVEF was calculated according to the recommendations by the American Society of Echocardiography.^23^

##### ECG and Signal‐Averaged ECG (SAECG)

Each participant in sinus rhythm underwent a resting supine 12‐lead ECG recording at 25 mm/s and a SAECG (MAC 5000 GE Medical, Milwaukee,WI, USA) by use of three X,Y,Z orthogonal bipolar leads (filter: 40‐250 Hz). Conventional criteria for the presence of late potentials were used (filtered QRS [fQRS]: ≥114 ms, low amplitude signal [LAS]: ≥38 ms, root mean square voltage [RMS]: ≤20 μV) for those with normal QRS duration,^19^, ^24^ but for patients with intraventricular conduction delay with a QRS duration ≥ 120 ms, the modified criteria (fQRS:≥145 ms, LAS:≥50 ms, RMS: ≤17.5 μV)^20^ were applied.

##### 24‐hour ambulatory ECG monitoring

During hospitalization, every patient in sinus rhythm underwent a 24‐hour AECG (Spider View). The recordings were analyzed using SyneScope 3.10 software (SpiderView & Synescope 3.10, Sorin Group, Clamart, France). The events were reviewed and manually corrected after having been automatically classified. Heart rate, RR intervals, number of ventricular premature beats (VPBs), and non‐sustained ventricular tachycardia (NSVT) episodes were calculated from the AECG.^25^

#### 2.2.2 Second step: Programmed ventricular stimulation

**FIGURE 1:**
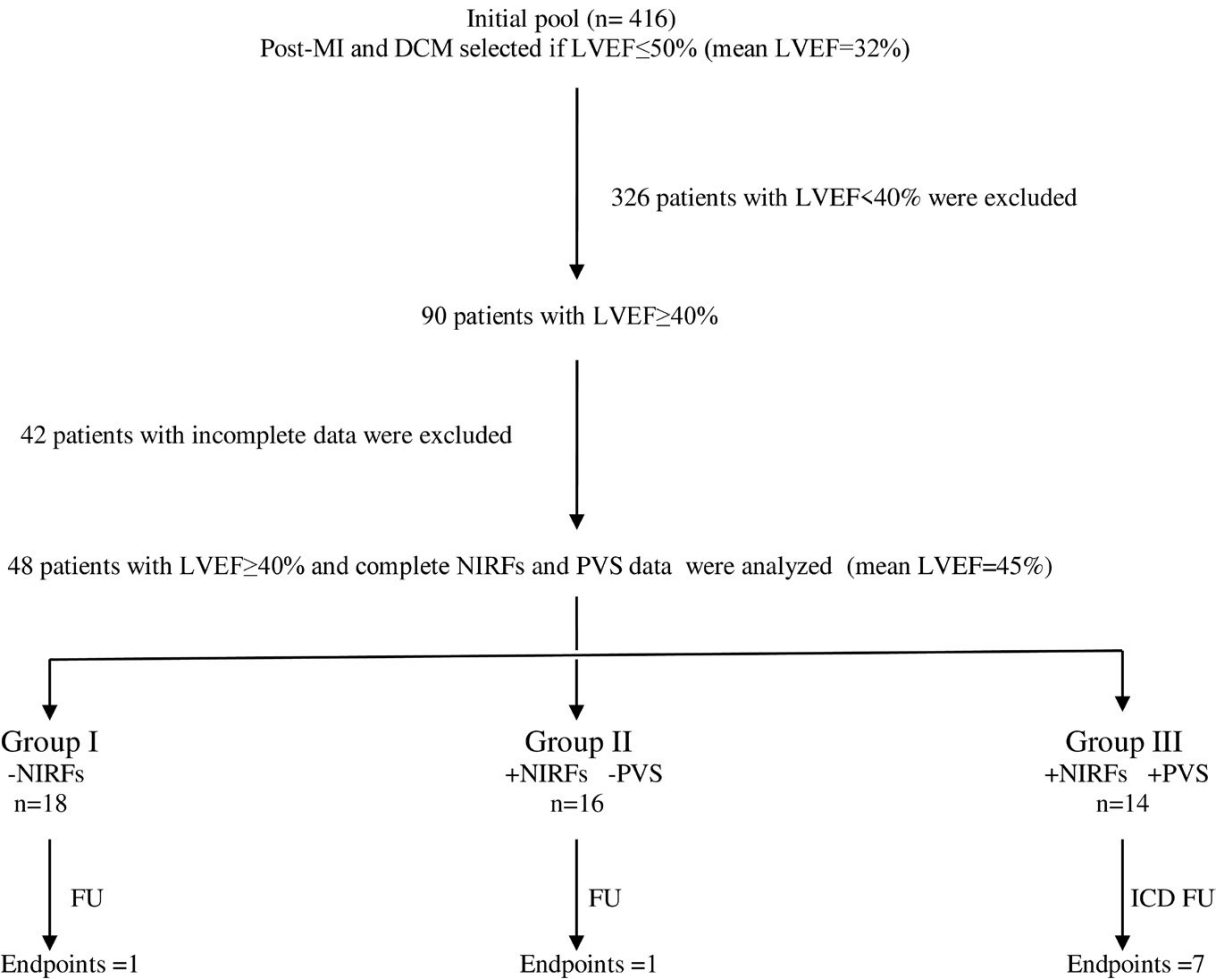
Flow chart of study and outcomes

After informed consent was obtained, NIRFs positive patients underwent PVS with three extrastimuli from two different right ventricle sites (Era‐His, Biotronik, Berlin, Germany). Stimulation protocol in our EP Lab has been described previously.^16^ DCM patients underwent additional PVS during iv isoproterenol infusion.^18^ The protocol was terminated prematurely if a sustained VT (monomorphic or polymorphic) or VF was induced. The combined two‐step algorithm is depicted in Figure 1.

##### Follow‐up

Follow‐up information was obtained either by having the patients visiting the outpatient arrhythmia clinic or by telephone contact. Patients with ICDs were seen at one month after discharge and then at 6‐month intervals throughout the follow‐up and whenever they reported palpitations or shocks. During each follow‐up visit, patients were examined and devices interrogated to determine the appropriateness of any delivered therapy (antitachycardia pacing and/or shocks). ICD therapies were classified appropriate when they occurred in response to VT or VF and inappropriate when triggered by supraventricular tachycardia, T‐wave over‐sensing, electrode dysfunction, or other environmental interactions of any cause. The study endpoints were the occurrence of a MAE either in the form of clinical VT/VF or/and appropriate ICD activation.

For the present study, stored data in our EP Lab were retrospectively analyzed. In the total database (n = 416), the mean LVEF was 32.4%. To investigate the HFmrEF subgroup, all patients with LVEF < 40% were excluded (n = 326). The remaining 90 patients were candidates for analysis. However, complete AECG, SAECG, and PVS data were available only for 48 of them. This subgroup (n = 48) was finally analyzed (Figure 1).

### 2.3 Statistical analysis

Continuous variables were reported as mean ± SD, with categorical variables as counts and percentages. The Shapiro‐Wilk test was applied to evaluate normality of the distributions. All reported p‐values are based on two‐sided tests and compared to a significance level of 5%. Sensitivity, specificity, predictive values and positive and negative likelihood ratios for the PVS were calculated. Univariate logistic regression analysis was performed for age, LVEF, NIRF variables, heart rate, and PVS. Multiple logistic regression analyses with the “enter” method were performed to identify the statistically significant predicting factors of MAE endpoint. Survival curves were estimated using the Kaplan‐Meier life tables and were compared with a log‐rank test. The power of the present study was post‐hoc calculated. Statistical analysis was performed with SPSS 25 (SPSS Inc, Chicago, IL) and Stata 13 (Stata, TX, USA).

## 3 RESULTS

The hospitalization cause of the target group of 48 HFmrEF patients included syncope (n = 7), acute ischemic episode (n = 14), HF deterioration (n = 12), serious supraventricular arrhythmic event (n = 4), and non‐sustained VT (n = 11) with some overlap existing between them. Hospitalization etiologies were not statistically different for the inducible vs the non‐inducible patients and the MAE positive vs the MAE negative subgroups. Most patients were males at the age of 65 ± 14 years with 69% of them suffering from CAD and the rest 31% suffering from DCM with the frequent concurrence of both hypertension and diabetes. Their mean LVEF was 45 ± 5%, and they were at an early New York Heart Association stage II. Both the fQRS and LAS on SAECG were relatively prolonged with late potentials detected in almost half of the population. There was also a mildy increased incidence of ventricular ectopy in the form of either frequent VPBs or/and NSVT runs. The patients clinicolabotary characteristics are presented in Table 1.

**TABLE 1:** Clinical characteristics and non‐invasive indices for total patients sample and G1, G2, and G3 groups

	All patients (n = 48)	G1 (n = 16) NIRF−	G2 (n = 18) NIRF+/PVS−	G3 (n = 14) NIRF+/PVS+	*P*‐value
Age (years)	64.92 ± 14.58	59.56 ± 16.50	65.83 ± 14.01	69.86 ± 11.61	.188
Male gender (%)	83.3	75	88.9	85.7	.572
CAD (%)	68.8	81.3	55.6	71.4	.541
DCM (%)	31.3	18.8	44.4	28.6	.541
Diabetes (%)	42.9	56.3	20	54.5	.287
Hypertension (%)	76.2	62.5	80	90.9	.181
LVEF (%)	45.69 ± 5.47	45.63 ± 6.29	46.11 ± 5.02	45.21 ± 5.40	.705
LVEDD (mm)	51.67 ± 5.28	51.08 ± 6.54	51.94 ± 4.63	51.92 ± 5	.836
NYHA (class)	2.0 ± 0.42	2.07 ± 0.46	1.83 ± 0.38	2.14 ± 0.36	.126
B blockers (%)	60.5	78.6	56.3	46.2	.206
ACEi/ARBs (%)	69	78.6	68.8	58.3	.277
Diuretics (%)	38.1	35.7	25	58.3	.088
Aspirin (%)	54.8	50	62.5	50	.695
Clopidogrel (%)	28.6	42.9	18.8	25	.531
Statins (%)	52.4	57.1	50	50	.845
Amiodarone (%)	11.9	21.4	0	16.7	.446
Std QRS (ms)	113.49 ± 23.07	102.33 ± 23.91	121.06 ± 21.80	115.71 ± 20.43	.396
fQRS (ms)	131.23 ± 25.69	117.53 ± 21.26	137.83 ± 20.10	137.43 ± 31.63	.415
LAS (ms)	49.17 ± 29.85	30.60 ± 9.35	49.72 ± 20.37	68.36 ± 41.5	.024
RMS (μV)	28.32 ± 21.54	39.60 ± 18.19	20.94 ± 13.07	25.71 ± 28.87	.096
2/3 LPs (%)	46.8	0	66.7	71.4	.028
VPBs (number)	4056 ± 6792.94	32.20 ± 56.03	4680.78 ± 6445.87	7833.77 ± 8825.54	.006
VPBs ≥ 240/24h (%)	47.8	0	61.1	84.6	.002
NSVT (number)	4.20 ± 17.05	0	9.28 ± 26.8	2 ± 2.61	.088
NSVT ≥ 1/24h (%)	30.4	0	44.4	46.2	.137
Heart rate (bpm)	70.89 ± 10.72	73.2 ± 12.18	69.11 ± 11.36	70.69 ± 7.96	.938

Note

*P*‐value denotes the p‐value comparing G3 vs G1 + G2.

Abbreviations: CAD, coronary artery disease; DCM, dilated cardiomyopathy; LVEF, left ventricular ejection fraction; LVEDD, left ventricular end diastolic diameter; NYHA, New YorkHeart Association class;ACEi, Angiotensin‐converting enzyme inhibitors; ARBs, Angiotensin II receptor blockers; std QRS:standard QRS; fQRS:filtered QRS; LAS, low amblitude signal; RMS:root mean square voltage; LPs, late potentials from SAECG; VPBs, ventricular premature beats number; NSVT, non‐sustained ventricular tachycardia episode(s) ≥1/24hour; PVS, programmed ventricular stimulation.

Sixteen of those 48 patients (Group 1, n = 16) were deemed to be of low risk based on the absence of NIRFs. The other 32 HF patients with at least one NIRF present were submitted to the invasive investigation with 18 non‐inducible on PVS (Group 2, n = 18) and 14 inducible on PVS (Group 3, n = 14) patients. Inducible patients received an ICD (n = 14) while Group 1 and Group 2 patients received only medical treatment. The G3 high‐risk patients did not differ from the low risk G1 and intermediate risk G2 patients in the underlying clinical status in terms of age, CAD, DCM etiology, presence of diabetes, and hypertension or the HF status. There was not a significant difference in the medications regimen used by the three groups of interest. However, the high‐risk G3 patients had longer LAS values in SAECG with more frequent detection of late potentials as well as a higher number of VPBs in 24‐hour AECG monitoring.

After 41 ± 18 months of follow‐up, nine patients experienced a MAE endpoint (clinical VT/VF = 3, appropriate ICD activation = 6). This endpoint occurred more frequently in Group 3 (50%) than in the rest of the patients (Group 1 & Group 2),(5.8%), (*P* = .001). One MAE endpoint occurred in a Group 1 patient after 55 months of follow‐up (male 61 years old, LVEF = 45%, with three vessels CAD and previous PTCA and CABG) succumbing to an irreversible sudden cardiac arrest one hour after the onset of severe chest pain in the context of an acute myocardial infarction upon his arrival in the emergency room. A Group 2 patient (male 46 years old, LVEF = 45%, with 1 vessel CAD and previous thrombolysis and PTCA) with positive late potendials in SAECG was also present with a MAE in the form of clinical VT.

**FIGURE 2:**
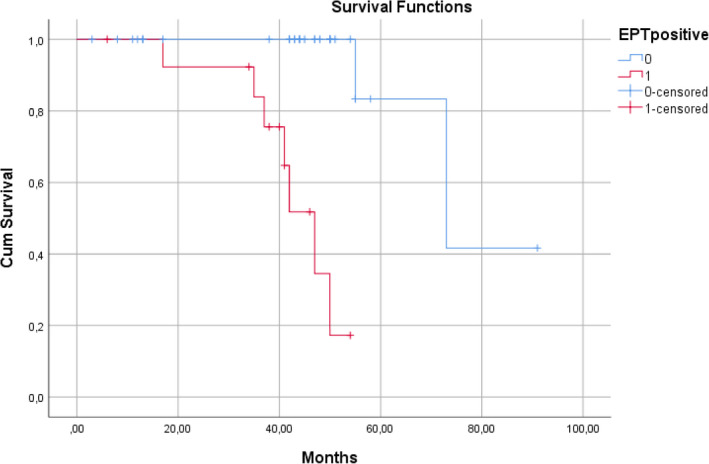
Kaplan‐Meier curve for MAE endpoint in G3 (positive electrophysiological test) vs G1 (NIRFs −) and G2 patients (NIRFs+ and PVS−)

In the univariate analysis only a positive PVS was significantly related with a MAE endpoint (Odds Ratio: 14.5, 95% CI: 2.457‐85.557, *P* = .003). No relationship was found regarding age, LVEF, and non‐invasive ECG, SAECG, and 24‐hour AECG‐derived prognostic variables (Table 2). A logistic multivariate regression model adjusted for age, LVEF, and PVS, revealed that PVS was an independent MAE predictor (OR: 21.152, 95% CI: 2.618‐170.887, *P* = .004) (Table 3). The PVS sensitivity was 87.5%, the specificity 70.8%, the positive predictive value 50%, and the negative predictive value 94.4%. The study flow with associated results is shown in Figure 1. The Kaplan‐Meier curves for the MAE end‐point in high vs moderate – low‐risk patients diverged significantly (log rank, *P* < .001) (Figure 2). The majority of events occurred after the first 2 years of follow‐up.

**TABLE 2:** Univariate analysis for the predictors of MAE

Predictors	Odds ratio	Confidence intervals	*P*‐value
Age	1.021	0.968‐1.078	.441
LVEF	1.091	0.953‐1.249	.205
LPs positive (2/3)	2.800	0.603‐13.011	.189
VPBs ≥ 240/24h	3.562	0.630‐20.155	.151
NSVT ≥ 1/24h	1.500	0.300‐7.491	.621
Heart rate	1.021	0.952‐1.095	.555
PVS +	14.5	2.457‐85.557	.003

Abbreviations: LVEF, left ventricular ejection fraction; LPs, late potentials from SAECG; VPBs ≥ 240/24h, ventricular premature beats number ≥ 240/24 hour; NSVT ≥ 1/24h, non sustained ventricular tachycardia episode(s) ≥1/24 hour; PVS, programmed ventricular stimulation.

**TABLE 3:** Multivariate analysis for the predictors of MAE

Variable	Odds ratio	Confidence intervals	*P*‐value
LVEF	1.153	0.966‐1.376	.114
PVS (+)	21.152	2.618‐170.887	.004
Age	0.996	0.930‐1.066	.906

Abbreviations: LVEF, left ventricular ejection fraction; PVS, programmed ventricular stimulation. The logistic multivariate regression model adjusted for PVS, age, and LVEF.

## 4 DISCUSSION

In this study, we retrospectively analyzed data from 48 hospitalized for a variety of reasons HFmrEF CAD and DCM patients with a mean LVEF = 45±5%, who were subsequently referred to our EP Lab for risk stratification for the primary prevention of SCD. We found that a multifactorial NIRFs leading to PVS two‐step approach was able to separate the high from the low‐risk HFmrEF population. We also provide evidence that these patients are adequately protected from MAEs by an ICD.

The causes for our HFmrEF patients hospitalization varied and they specifically included syncope, acute coronary syndrome, HF deterioration, supraventricular arrhythmia with haemodynamic destabilization and non‐sustained VT. Considering the hospitalization etiologies these patients presented with, they were not the typical community HFmrEF population but rather a hospitalized HFmrEF subgroup with a higher arrhythmic and mortality risk profile. These patients also suffered frequently from diabetes and hypertension. No statistical differences were observed between groups for comorbitidies.

We risk stratified them for SCD with a combined two‐step risk stratification approach. In the first‐ step during the non invasive screening, all the negative NIRFs patients deemed to be low risk (Group 1) while all the positive NIRFs patients were deemed to be of indermediate SCD risk. A further refinement of the risk for the later patients was achieved in the second‐step with an invasive programmed ventricular stimulation (PVS). Non‐inducible patients with at least one positive NIRF consisted the Group 2. Inducible patients deemed as high risk (Group 3) received an ICD. After 41 ± 18 months of follow up, nine patients experienced MAE endpoints. This endpoint occurred more frequently (50%) in Group 3 (inducible) than in Groups 1 and 2 (5.8%) (negative NIRFs patients and non‐inducible, respectively, *P* = .001). PVS in multivariate analysis was found to be an independent MAE predictor with odds ratio of 21. Our results confirm the efficacy of a two‐step approach in SCD risk stratification previously reported in studies investigating depressed left ventricular systolic function populations^26^, ^27^ for our post‐MI and DCM HFmrEF patients as well. The power of the present study with 48 patients (Group 3, n = 14 vs Groups 1 and 2, n = 34) and 7 and 2 MAE for the high and the low/intermediate risk groups, respectively, retrospectively was found to be 0.90.

The selection of LVEF as a classification tool for ominous echocardiographic phenotypes of heart failure (HFrEF – HFmrEF – HFpEF) has been chalenged recently.^28^ However the LVEFcut‐off point of 35% remains the principal risk stratification tool for the primary prevention of SCD with current guidelines recommending an ICD only for the post‐MI and DCM patients with LVEF ≤ 35%.^8^, ^15^ The selection of this cut‐off point as a marker of risk, treat the arrhythmic SCD risk as a binary state. This selection reflects the MADIT II^29^ and SCD‐HeFT^30^ trials study design. Patients with a LVEF > 35% were ignored and this happened not because their SCD risk was insignificant, but rather from the nessecity for focusing in a subpopulation in which more frequent endpoints were expected. In fact, SCD risk is continuous through the entire LVEF spectrum with a reverse trend, the more LVEF increases the more SCD decreases, but it never abolishes even in patients with a LVEF > 50%.^6^, ^31^, ^32^, ^33^ Arrhythmic SCD risk in such patients with high LVEF may originate from fibrotic scar areas of small previous myocardial infarctions or silent myocardial infarctions even without a clinical history of CAD.^34^ Nowadays we acknowledge the need for protecting the HFmrEF and HFpEF patients as well,^13^ which is a larger population on an epidemiological basis.^35^ Populations with a relatively preserved LVEF were explored only in a few previous studies. In post‐MI patients with an average LVEF = 55±10%, the presence of T‐wave alternans, non‐sustained VT or/and ventricular late potentials intentified a subpopulation at risk.^6^ In ISAR‐Risk study, an abnormal heart rate turbulence (HRT) and deceleration capacity (DC) of the heart rate identified a subgroup with severe autonomic failure that had a high mortality risk.^36^ Current studies attempt to select high‐risk patients for an ICD implantation based on the presence of NIRFs (REFINE‐ICD)^37^ and Magnetic Resonance Imaging findings (GUIDE‐CMR).^38^ The PRESERVE‐EF study^13^ recently published results, based on a 7 NIRFs first‐step algorithm, confirming the efficacy of a multifactorial EP inclusive two‐step approach for post MI patients with LVEF > 40%, pointing to the presence of a rather small but significant subgroup of high‐risk post‐MI patients at an early mostly asymptomatic HF stage which could be protected by an ICD. The present single‐center study screened patients between 2005 and 2013, while the PRESERVE EF study – a 7‐center, collaborative study – screened patients at a later time. These two studies are different in terms of time of execution, patient characteristics, and results. The present single‐center study included CAD and DCM patients with a mean LVEF = 45% at a mean NYHA II providing results for 3 NIRFs approach, while the multicenter PRESERVE EF study enrolled post‐MI patients with a mean LVEF = 50.8% mostly at a NYHA I providing results for 7 NIRFs. A two‐step risk stratification using 3‐NIRFs approach for patient selection for PVS was used by our EP Lab for the past 20 years, and the results for primary prevention of SCD have been published for CAD,^16^ DCM^18^ and Hypertrophic Cardiomyopathy^39^ patients. Based on the progress of our research as well as on all the latest advances on Holter monitor technology that was built up by the advanced AECG software readily available at the time, we decided, in 2014, to incorporate complementary NIRFs (QTc, SDNN, TWA, HRT/DC) into the PRESERVE EF study protocol for the quantification of the presence and activity of additional mechanisms of arrhythmogenesis (QTc and TWA for the prolonged and unstable repolarization; SDNN and HRT/DC for the impaired autonomic nervous status with enhanced sympathetic and suppressed parasympathetic activity). While a 7‐NIRF first screening step for the intermediate arrhythmic risk patients detection undoubtedly provides enhanced sensitivity, at the sametime requires a higher number of them to undergo the second PVS step. A satisfactory level of risk stratification can also be achieved, even with the simplified 3‐NIRF approach including late potentials presence from SAECG, VPBs ≥ 240/24h and NSVT ≥ 1/24h from AECG, as previous^26^, ^27^ as the present studies clearly demonstrate. Thus, provided that the specific Ambulatory ECG software is available, we suggest the 7‐NIRF approach; but, for EP Labs and Cardiology Departments where such advanced software is not currently available, we suggest the 3‐NIRF simplified algorithm, which is possible to be easily extracted from the conventional AECG and SAECG. Post‐MI and DCM HFmrEF patients at risk for arrhythmic SCD is a specific subpopulation. For the detection of this subpopulation a two‐step approach may be proven adequate and sufficient.^40^, ^41^ NIRFs presence investigation in the first‐step, is applicable to everyone, and diminishes significantly the number of patients in need for further invasive screening. NIRFs reflect the presence and activity of different arrhythmogenesis mechanisms. Late potentials are related to post‐MI fibrotic scars that may form reentrant circuits while VPBs and NSVT reflect both the presence of arrhythmogenic substrate and triggered activity. PVS reveals the vulnerability of such an impaired myocardial substrate for the future event of malignant ventricular arrhythmias. This combination is well applicable to HFmrEF subpopulation for selection of the appropriate patients who may benefit from an ICD implantation.

### Limitations of the study

The study is based on restrospective analysis of a limited number of HFmrEF patients screened for primary prevention of SCD in our EP Lab. According to their hospitalization etiology, these 48 patients represent probably a higher arrhythmic risk profile subgroup of the total HFmrEF population. Thus, our observed inducibility on PVS and MAE event rates are probably not reflecting the risk existing in the broader HFmrEF population in the community. It is likely that the incidence of positive NIRFs as well as the inducibility on PVS rates might have been higher in our seriously affected hospitalized population subgroup of HFmrEF patients, compared to the rest of the corresponding community population. While it is known that beta‐blockers may protect against malignant arrhythmias, a significant part of our patients was undertreated, with only 60% of them receiving beta‐blockers and 69% receiving angiotensin‐converting‐enzyme inhibitors or angiotensin II receptor blockers. However, it should be noted that no significant difference in therapies for the high risk (G3) vs the low and intermediate risk (G1 and G2) groups have been observed. Furthermore, among the examined NIRFs we did not include novel indices like DC, HRT, T‐wave alternans, QT interval duration, or modern Imaging modalities from Echocardiography and Magnetic Resonance of the Heart.

## 5 CONCLUSIONS

A combined NIRFs guiding to PVS, two‐step risk stratification approach, detected efficiently those post‐MI and DCM Heart Failure mid‐range Ejection Fraction (HFmrEF/40%‐49%) hospitalized patients, to be at risk for arrhythmic SCD.

## CONFLICTS OF INTEREST

Authors declare no conflict of interests for this article.
